# Femoral nailing associated with bone marrow emboli in pigs induced a specific increase in blood IL-6 and broad inflammatory responses in the heart and lungs

**DOI:** 10.3389/fimmu.2024.1396800

**Published:** 2024-07-19

**Authors:** Steinar Kristiansen, Benjamin Stage Storm, Åse Eeg Emblem, Renathe Henriksen Grønli, Kristin Pettersen, Jonas Hilmo, Anders Hagen Jarmund, Martin Leth-Olsen, Siri Ann Nyrnes, Bent Aksel Nilsen, Erik Waage Nielsen, Tom Eirik Mollnes

**Affiliations:** ^1^ Department of Surgery, Nordland Hospital, Bodø, Norway; ^2^ Institute of Clinical Medicine, University of Tromsø, Tromsø, Norway; ^3^ Faculty of Nursing and Health Sciences, Nord University, Bodø, Norway; ^4^ Research Laboratory, Nordland Hospital, Bodø, Norway; ^5^ Department of Circulation and Medical Imaging, Faculty of Medicine and Health Sciences, Norwegian University of Science and Technology, Trondheim, Norway; ^6^ Children’s Clinic, St. Olavs Hospital, Trondheim University Hospital, Trondheim, Norway; ^7^ Department of Pain Medicine and Research, University of Oslo and Oslo University Hospital, Oslo, Norway; ^8^ Department of Immunology, University of Oslo and Oslo University Hospital, Oslo, Norway

**Keywords:** bone marrow embolization, fat embolism syndrome, inflammation, orthopedic surgery, complement, cytokines

## Abstract

**Introduction:**

Bone marrow embolization may complicate orthopedic surgery, potentially causing fat embolism syndrome. The inflammatory potential of bone marrow emboli is unclear. We aimed to investigate the inflammatory response to femoral intramedullary nailing, specifically the systemic inflammatory effects in plasma, and local tissue responses. Additionally, the plasma response was compared to that following intravenous injection of autologous bone marrow.

**Methods:**

Twelve pigs underwent femoral nailing (previously shown to have fat emboli in lung and heart), four received intravenous bone marrow, and four served as sham controls. Blood samples were collected hourly and tissue samples postmortem. Additionally, we incubated bone marrow and blood, separately and in combination, from six pigs *in vitro*. Complement activation was detected by C3a and the terminal C5b-9 complement complex (TCC), and the cytokines TNF, IL-1β, IL-6 and IL-10 as well as the thrombin-antithrombin complexes (TAT) were all measured using enzyme-immunoassays.

**Results:**

After nailing, plasma IL-6 rose 21-fold, compared to a 4-fold rise in sham (p=0.0004). No plasma differences in the rest of the inflammatory markers were noted across groups. However, nailing yielded 2-3-times higher C3a, TCC, TNF, IL-1β and IL-10 in lung tissue compared to sham (p<0.0001-0.03). Similarly, heart tissue exhibited 2-times higher TCC and IL-1β compared to sham (p<0.0001-0.03). Intravenous bone marrow yielded 8-times higher TAT than sham at 30 minutes (p<0.0001). *In vitro*, incubation of bone marrow for four hours resulted in 95-times higher IL-6 compared to whole blood (p=0.03).

**Discussion:**

A selective increase in plasma IL-6 was observed following femoral nailing, whereas lung and heart tissues revealed a broad local inflammatory response not reflected systemically. *In vitro* experiments may imply bone marrow to be the primary IL-6 source.

## Introduction

Fat embolism syndrome following fractures and orthopedic surgery is caused by bone marrow emboli, consisting of cell-rich (red), and fat-rich (white) bone marrow. Bone marrow cells including mesenchymal stem cells and hematopoietic cells can produce a large number of cytokines, including IL-6 and TNF (1–3).

Bone marrow emboli can cause infarcts in the lung, heart, and brain, precipitating fat embolism syndrome (4, 5). While the clinical definition of fat embolism syndrome varies (4), we consider that organ failure occurring after verified bone marrow embolization may represent fat embolism syndrome.

Asymptomatic bone marrow emboli occur frequently after trauma and orthopedic surgery, fat embolism syndrome in far fewer cases (6). The incidence of fat embolism syndrome after orthopedic trauma varies between 1-3% in patients with a single fracture in a long bone and may be as high as 30% in patients with fractures in multiple long bones (6). The condition is most common in the young, while mortality is up to 30% in older patients with comorbidities (7).

The systemic inflammatory response constitutes a central aspect of the pathophysiology of fat embolism syndrome (7). Whether the inflammatory response arises due to tissue trauma and surgery or is triggered by bone marrow emboli remains unclear (8). However, particularly IL-6 increases following intramedullary nailing of the femur (9, 10), and elevated IL-6 is associated with fat embolism syndrome (11). Elevated IL-6 levels are linked with increased morbidity and mortality following both orthopedic trauma and elective surgery (12, 13).

Consequently, we aimed to examine the scope of inflammation induced by intramedullary nailing of the femur in a porcine model. We recently reported bone marrow emboli to the lungs and heart after femoral nailing in 11 of 12 animals studied (14). In the present study we investigated the systemic response in blood and local inflammatory responses in lung and heart, with emphasis on complement activation and cytokine release in these animals. Additionally, the plasma response was compared to that from autologous bone marrow injections in a separate set of animal experiments. Finally, since IL-6 is produced by various cell lines in different tissues (15), we sought to determine the extent to which bone marrow alone contains or produces this cytokine in a porcine *in vitro* model.

## Materials and methods

### Animals

**Figure 1:**
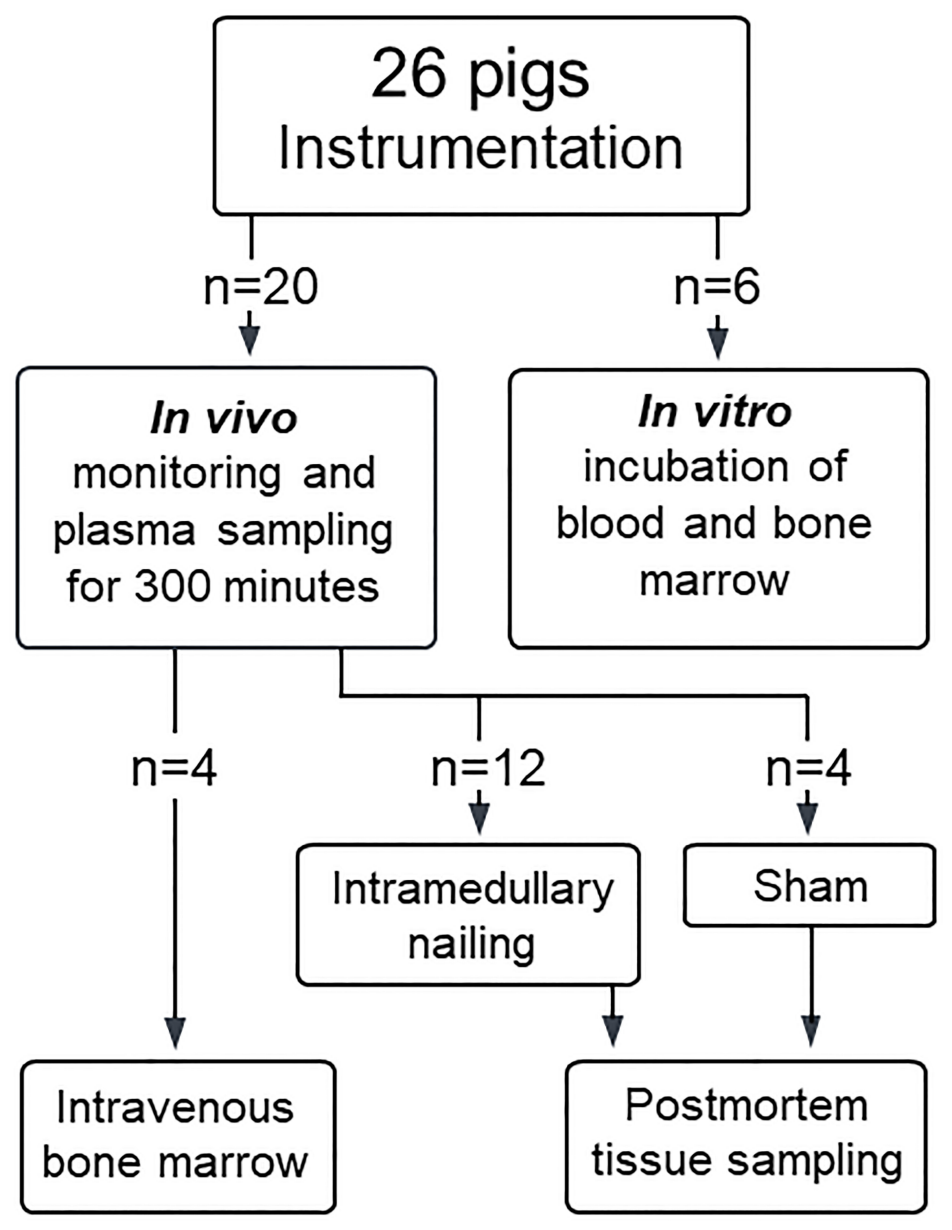
Allocation of pigs. A total of 26 pigs were included, of which 20 were allocated to the *in vivo* experiments and 6 for *in vitro* experiments. Of the 20 pigs undergoing *in vivo* experiments, 12 underwent bilateral intramedullary nailing of the femur, four were exposed to intravenous injection of autologous bone marrow and four served as sham, undergoing instrumentation, monitoring, and sampling only. Postmortem tissue samples were obtained only from pigs undergoing intramedullary nailing and sham.

The study was approved by the Norwegian Animal Welfare Committee (FOTS ID 19803) and conducted in accordance with the Norwegian regulations for laboratory animal care and EU directive 2010/63/EU. Included pigs were specific pathogen-free Norwegian landrace pigs with an average weight of 27 (SD 4) kg, of which 12 underwent intramedullary reaming and nailing, four intravenous injection of autologous bone marrow, and four were sham-operated controls. The 12 pigs undergoing intramedullary nailing has been published recently, with data on fat emboli deposited in lung, heart and brain (14). Blood and tissue samples from these animals were used for analyses in the present study. Bone marrow and blood were collected from an additional six pigs (average weight 42 (SD 7) kg) for *in vitro* experiments (**Figure 1**). Exclusion criteria were pre-existing illness, procedural complications unrelated to intramedullary nailing and open foramen ovale. No pigs were excluded.

### Instrumentation, anesthesia, and monitoring

The animals were handled as previously described (14). Briefly, the pigs were anesthetized using intramuscular azaperone, ketamine, and intravenous pentobarbital until endotracheal intubation was achieved. Anesthesia was maintained with morphine, midazolam, and pentobarbital. Minute ventilation was titrated to a pH of 7.4 ± 0.5, and inspired oxygen (FiO_2_) adjusted to maintain arterial pulse oximetry saturation (SpO_2_) above 90%.

To avoid coagulation of the intravascular catheters, a 3 mL/h infusion of saline-heparin flush solution (2.5 IU heparin/mL) was administered to the intra-arterial and intravenous catheters We recorded 6-lead ECG with ST-segment changes, SpO_2_, end-tidal CO_2_, continuous invasive arterial and central venous pressures (IntelliVue MP70, Phillips, Amsterdam, Netherlands).

The pigs were assigned to one of three groups: bilateral intramedullary nailing of the femur (n = 12), intravenous administration of bone marrow (n = 4), or sham-operated controls (n = 4). A total amount of 100 mL of bone marrow was aspirated bilaterally using the Arrow EZ-IO intraosseous vascular access system (Teleflex, Wayne, PA) from the femur (hind legs) of the pigs and immediately injected intravenously in a 14G Venflon venous catheter (BD, Franklin Lakes, NJ) placed in an ear vein over an average time period of 30 minutes. The average duration of intramedullary nailing was approximately 30 minutes. After approximately 300 minutes of observation, we euthanized the pigs via central venous injection of potassium chloride. Additionally, six pigs underwent identical anesthesia and instrumentation before arterial blood sampling and bone marrow sampling for *in vitro* experiments.

### Blood and tissue sampling and analysis

Blood was collected from the carotid artery upon placement of the arterial catheter approximately 60 minutes prior to any intervention, immediately before either surgery or intravenous administration of bone marrow or 30 minutes after completed instrumentation in sham-operated controls, and after two-, four- and approximately five-hours observation.

During the experiments, we sampled a total of 75 mL of blood per animal, using Vacutainer closed vacuum system and serum tubes with clot activator and gel (Vacuette, Greiner Bio-One GmbH, Kremsmünster, Austria) for serum samples, and safePICO aspirator blood gas syringe with heparin (Radiometer Medical ApS, Brønshøj, Denmark) for arterial blood gas analysis. Blood sampling was performed as described by Storm (16) in order to avoid pre-analytical heparin contamination from the line flush solution. After 30 minutes of clotting, we centrifuged the serum tubes at 2000 g for 10 minutes. The serum was then transferred to cryotubes and stored at -80°C for analysis. Arterial blood gases were analyzed immediately after sampling. A set of EDTA tubes were stored at room temperature for up to 8 hours and analyzed for white blood count using the ADVIA 2120i (Siemens Healthcare GmbH, Erlangen, Germany) or IDEXX ProCyte Dx (IDEXX Laboratories, Westbrook, ME). A set of EDTA tubes were immediately centrifuged at 4°C at 1500 g for 15 min and plasma isolated and frozen at -80°C for later analysis of complement and cytokines as described below.

Troponin I was analyzed on the Atellica IM analyzer (Siemens Healthineers, Siemens Healthcare GmbH, Erlangen, Germany). Arterial blood gas analysis was performed using an ABL 80 Flex blood gas analyzer (Radiometer Medical ApS, Brønshøj, Denmark).

Lung and heart tissue was sampled from the operated pigs and sham-operated controls. The samples were snap-frozen on dry ice in Nunc tubes (Thermo Scientific, Roskilde, Denmark) with no additive for further homogenization and analysis.

### Homogenization of lung and heart tissue

For complement analysis, approximately 100 mg of tissue was transferred to gentleMACS M-tubes (Miltenyi Biotec, Bergisch Gladbach, Germany), and a mixture of 10 μL Protease Inhibitor Cocktail Set I (Merck KGAA, Darmstadt, Germany) and 1 mL CytoBuster Protein Extraction Reagent (Millipore Sigma, Burlington, MA) was added to the samples, and the samples were homogenized using profile 7 (gradient homogenization intervals with increasing speeds up to 4000 rpm with short backward spins and a total run time of 30 seconds) on the Medic Tools Dispomix Drive (Miltenyi Biotec). After homogenization, the samples were incubated for 5 minutes on ice, centrifuged for 20 minutes at 2500 g at 4°C, and the supernatant was transferred to 1 mL Matrix tubes (Thermo Fisher Scientific) and stored at -80°C for later analysis.

### Analysis of complement in plasma, lung and heart tissue

We measured complement C3a using ELISA with porcine-specific C3a monoclonal antibodies as previously described (17). The antibody binds to a neoepitope exposed when C3a is cleaved off C3, and the assay only detects free C3a in the fluid phase. We measured the soluble terminal C5b-9 complex (terminal complement complex, TCC) using ELISA with the anti-human-C9 neoepitope antibody clone aE11 produced in-house as capture antibody and a porcine cross-reacting anti-human C6 (Quidel, San Diego, CA) as detection antibody as described in detail (18, 19). It is previously documented that the aE11 cross-reacts with porcine TCC (18).

### Analysis of cytokines in plasma

We analyzed EDTA plasma for the following cytokines using immunoassays: Tumor necrosis factor (TNF) and interleukin (IL)-6 using the Porcine TNF and IL-6 Quantikine sandwich ELISA kit (R&D Systems Inc, Minneapolis, MN) with optical density measured by Infinite M200 Pro microplate reader (Tecan Trading AG, Switzerland); IL-1β using porcine MILLIPLEX map Kit (Merck, EMD Millipore Corporation, Billerica, MA) and IL-10 using Invitrogen ProcartaPlex Multiplex Porcine Immunoassay (Bender MedSystems GmbH, Vienna, Austria), and the fluorescence intensity analyzed on a Bio-Plex 200 Multiplex Analyzer (Bio-Rad Laboratories, Gurugram, India). All analyses were performed in accordance with the manufacturer’s instructions.

### Analysis of cytokines in lung and heart tissue

We analyzed tissue samples for TNF and IL-1β using a Quantikine sandwich ELISA kit (R&D Systems Inc), IL-6 using a porcine Luminex Discovery Assay (R&D Systems Inc) and IL-10 using Invitrogen ProcartaPlex Multiplex Immunoassay for Porcine assay (Bender MedSystems GmbH, Vienna, Austria). The fluorescence intensity was analyzed on a Bio-Plex 200 Multiplex Analyzer (Bio-Rad Laboratories). All analyses were performed in accordance with the manufacturer’s instructions. Results are given pr. mL homogenate.

### Analysis of thrombin-antithrombin complex in plasma

We quantified the thrombin-antithrombin complex (TAT) in EDTA plasma using the human Enzygnost TAT micro (Siemens Healthcare Diagnostics Products GmbH, Marburg, Germany), in accordance with the manufacturer’s instructions. Cross-reactivity to porcine TAT has been documented (20). The optical density was measured using an Infinite M200 Pro microplate reader (Tecan Trading AG).

### *In vitro* experiments

In the six pigs used for *in vitro* experiments, 4 mL arterial blood was collected approximately 30 minutes after instrumentation was completed. 10 mL of bone marrow was aspirated simultaneous to blood sampling, by insertion the Arrow EZ-IO intraosseous vascular access system (Teleflex, Wayne, PA) in the hind leg. Lepirudin was used to prevent coagulation, with a concentration in sampled arterial blood of 0.05 mg/mL and 0.1 mg/mL in bone marrow.

Bone marrow was homogenized using gentleMACS M-tubes and Medic Tools Dispomix Drive (Miltenyi Biotec) at profile 7. Samples of either 800 µL of blood, 400 µL blood mixed with 400 µL bone marrow, or 800 µL of bone marrow was centrifuged at 3000 x *g* for 20 minutes at 4°C either directly or after incubation on a Rock’n’Roller tube roller mixer (Labinco, Breda, NL) at 37°C for two or four hours. EDTA (final concentration 10mM) was added to all samples before centrifugation. Plasma samples were stored at -80°C until analysis.

### Statistical analysis

We performed statistical analysis using GraphPad Prism, version 10 (GraphPad Software Inc., San Diego, CA). Data are presented as mean and 95% confidence interval (CI). Lower confidence interval limit for biological data, where true mean cannot be below zero, was bounded at zero. When comparing only two groups, we analyzed group differences using unpaired, two-tailed Student’s t-test for normally distributed data, or the Mann-Whitney test for not normally distributed data. We tested for normality using the Anderson-Darling and the Shapiro-Wilk test. When comparing three groups, we used the one-way ANOVA test or the restricted maximum likelihood mixed model (REML). When comparing difference over time in the same group, the one-sample t-test was used. p < 0.05 was considered statistically significant.

## Results

The 12 animals undergoing intramedullary nailing have been described in detail previously, including the deposition of fat emboli in the lung and the heart (14).

### Baseline characteristics

The baseline conditions for the 20 animals included in the present study are summarized in **Table 1**.

**Table 1:** Baseline characteristics of 20 pigs divided into three groups: intramedullary nailing (n=12), intravenous bone marrow infusion (n=6) and sham (n=4).

		Intramedullary nailing	Intravenous bone marrow	Sham	p^1^
Weight	kg	29 (27 - 31)^1^	29 (25 - 32)	17 (5 - 11)	0.001
WBC	K/µL	17 (15 - 19)	15 (13 - 16)	14 (13 - 15)	0.001
C3a	ng/mL	29 (21 - 37)	20 (5 - 36)	26 (3 - 50)	>0.05
TCC	CAU/mL	0.6 (0.5 - 0.8)	0.8 (0.2 - 1.4)	0.7 (0.4 - 0.9)	>0.05
IL-6	pg/mL	4.1 (2.9 - 5.4)	4.7 (2.5 - 6.9)	9.4 (0.1 - 21)	0.0004
TAT	ng/mL	72 (51 - 92)	49 (32 - 66)	51 (14 - 89)	<0.0001

^1^Mean values with 95% confidence intervals are reported with p-value for difference between groups using mixed-models REML analysis. WBC, white blood cells; CAU, complement arbitrary units; TAT, thrombin-antithrombin complexes.

### Complement, cytokines, and coagulation in plasma

**Figure 2:**
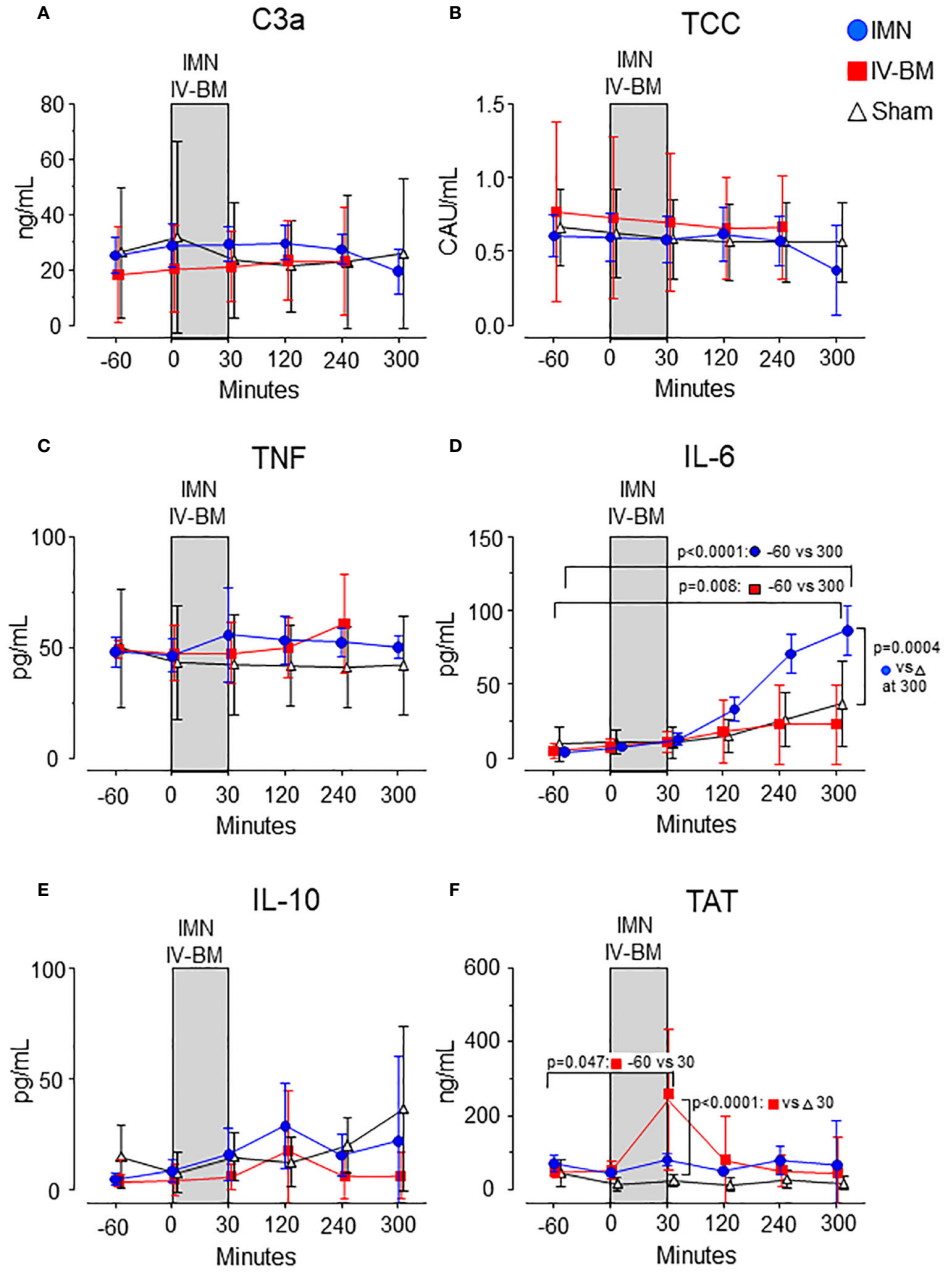
Complement activation products, cytokines, and TAT in plasma. C3a **(A)**, TCC **(B)**, TNF **(C)**, IL-6 **(D)**, IL-10 **(E)**, and TAT **(F)** were measured in the groups exposed to intramedullary nailing (IMN, blue circles), intravenous bone marrow (IV-BM, red squares) or sham (open triangles), and analyzed for the inflammatory mediators during and observation period of 300 minutes. The gray field spanning from 0 to 30 minutes represents the approximate average duration of either intramedullary nailing (IMN) or injection of intravenous bone marrow (IV-BM). Data are presented as mean values, with error bars spanning the 95% confidence interval (CI). Baseline values compared to values after 300 minutes were analyzed using the one-sample t-test **(D, F)**. Differences between groups were analyzed using the unpaired t-test.

In intramedullary nailed pigs, plasma IL-6 increased from 4.1 (95% CI 2.9 - 5.4) pg/mL at baseline to 87 (95% CI 70 - 103) pg/mL 300 minutes after start of surgery (p<0.0001) (**Figure 2D**). In sham animals an increase from 9.4 (95% CI 0 - 21) pg/mL to 37 (95% CI 8 - 65) was observed during the same time period (**Figure 2D**). The increase was significantly higher in intramedullary nailed pigs compared to sham (p=0.0004) (**Figure 2D**). No significant differences were observed for plasma C3a, TCC, TNF, IL-10 and TAT (**Figures 2A–C, E, F**).

In pigs exposed to intravenous bone marrow, IL-6 increased from 5 (95% CI 2 - 7) pg/mL at baseline to 23 (95% CI 0 - 50) pg/mL 300 minutes after injection (p=0.008) but did not differ significantly compared to sham (**Figure 2D**). Plasma C3a, TCC, TNF and IL-10 did not increase nor differ significantly between pigs exposed to intravenous bone marrow compared to sham (**Figures 2A–C, E**).

In pigs exposed to intravenous bone marrow, TAT increased from 49 (95% CI 32 - 66) ng/mL at baseline to 244 (95% CI 51 - 436) ng/mL 30 minutes after injection (p=0.047) (**Figure 2F**), 8 times higher than sham (p<0.0001).

Except for this increase in TAT, C3a, TCC, TNF, IL-6 and IL-10 remained at baseline levels throughout the observation period (**Figures 2A–E**). No significant changes were seen for any of these thromboinflammatory markers in the sham pigs (**Figures 2A–F**).

### Complement and cytokines in tissue

**Figure 3:**
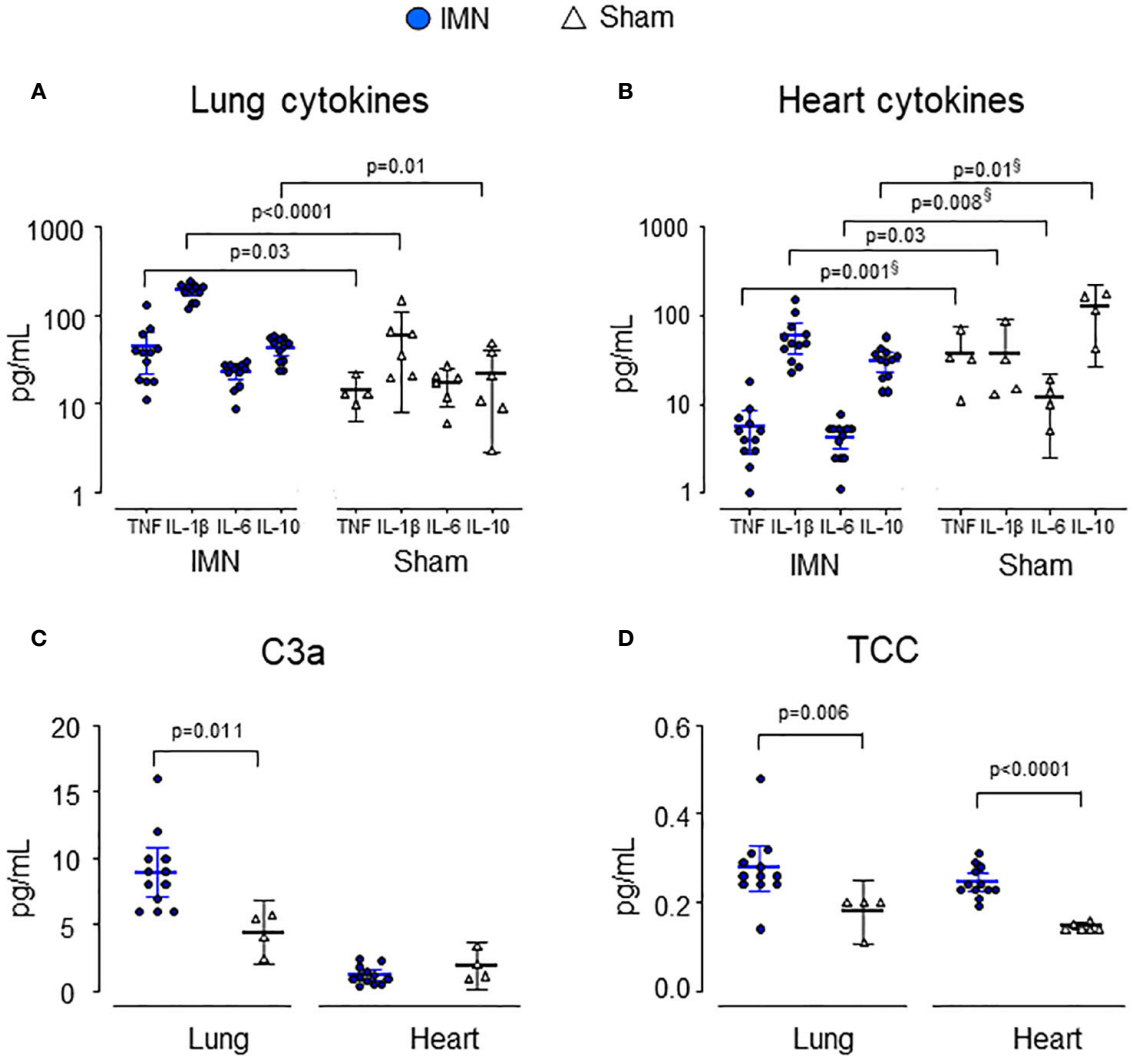
Cytokines and complement activation products in lung **(A, C)** and heart **(B, D)** tissue. Lung and heart cytokines and complement products are depicted from intramedullary nailed pigs (IMN) (blue circles) compared to sham (open triangles). Data are presented as mean values, with error bars spanning the 95% confidence interval (CI). P-values are reported after testing with the Mann-Whitney Test. Where there was a significant difference between groups but lower mean values in operated pigs vs sham, p-values are marked with^§^.

In lung tissue, C3a, TCC, TNF, IL-1β and IL-10 were all significantly higher in intramedullary nailed vs sham (**Figures 3A, C, D**). C3a was 8.9 (95% CI 7.1 - 10.8) ng/mL in intramedullary nailed pigs vs 4.4 (95% CI 2.0 - 6.8) ng/mL in sham (p=0.011), TCC 0.3 (95% CI 0.2 – 0.3) CAU/mL in intramedullary nailed pigs vs 0.2 (95% CI 0.1 - 0.3) CAU/mL in sham (p=0.006), TNF 43 (95% CI 22 - 64) pg/mL in nailed pigs vs 13 (95% CI 10 - 16) in sham (p=0.03), IL-1β 189 (95% CI 165 - 214) pg/mL in intramedullary nailed pigs vs 58 (95% CI 8 - 109) in sham (p<0.0001) and IL-10 43 (95% CI 34 - 51) pg/mL in intramedullary nailed pigs vs 21 (95% CI 3 - 40) pg/mL in sham (p=0.01).

In heart tissue, only IL-1β and TCC were significantly higher in intramedullary nailed pigs vs sham (**Figures 3B–D**). TCC was 0.3 (95% CI 0.2 - 0.3) CAU/mL vs 0.2 (95% CI 0.1 - 0.2) CAU/mL in sham (p<0.0001) and IL-1β 60 (95% CI 37 - 83) pg/mL in intramedullary nailed pigs vs 29 (95% CI 0 - 59) pg/mL in sham (p=0.03).

### Oxygenation, mean arterial pressure and troponin I

**Figure 4:**
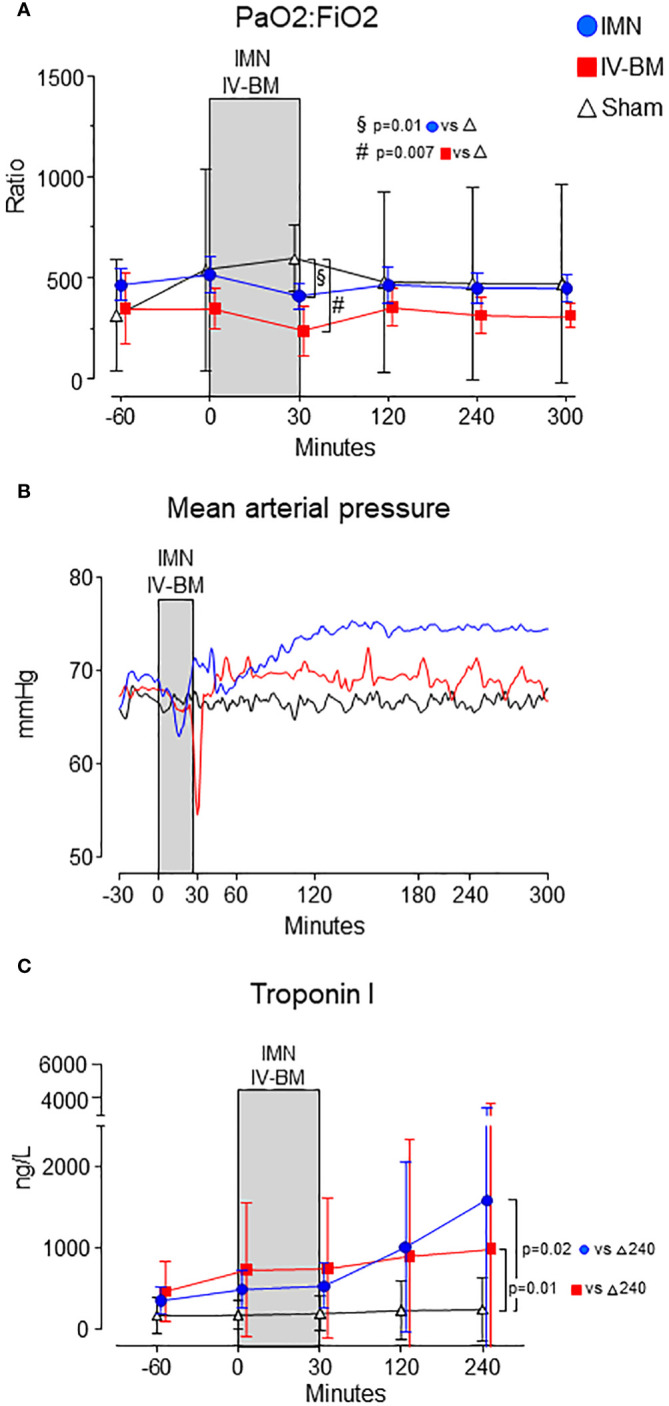
Cardiopulmonary parameters. PaO2:FiO2 ratio **(A)**, mean arterial pressure **(B)** and Troponin I **(C)** are depicted from intramedullary nailed pigs (IMN), pigs injected with intravenous bone marrow (IV-BM) and sham. The gray field spanning from 0 to 30 minutes represents the approximate average duration of either intramedullary nailing (IMN) or injection of intravenous bone marrow (IV-BM). In **(A, C)** data are presented as mean values, with error bars spanning the 95% confidence interval (CI). In **(B)** mean values are represented by continuous lines. Unpaired t-tests were performed, comparing intramedullary nailed pigs and sham, and pigs intravenously injected with bone marrow and sham, respectively, at 30 minutes after surgery or injection.

Intravenous injection of bone marrow caused a pronounced hypoxia and hypotension, but mild hypoxia and hypotension also occurred in pigs undergoing intramedullary nailing, as compared to sham (**Figures 4A, B**). Intramedullary nailing caused highest troponin I values (**Figure 4C**).

Compared to sham, PaO_2_/FiO_2_-ratio were reduced 30 minutes after nailing or injection in both intramedullary nailed pigs (-184 (95% CI -318 - -55) mmHg) (p=0.011) and after bone marrow injection (-357 (95% CI -424 - -88) mmHg) (p=0.007) (**Figure 4A**).

In intramedullary nailed pigs, troponin I at 240 minutes was 1580 (95% CI 0 - 3456) ng/L vs 241 (95% CI 0 - 625) ng/L in sham (p=0.019), and 981 (95% CI 0 - 2806) ng/L in pigs exposed to intravenous bone marrow and 241 (95% CI 0 - 625) ng/L, also higher vs sham (p=0.012) (**Figure 4C**).

### *In vitro* experiments

**Figure 5:**
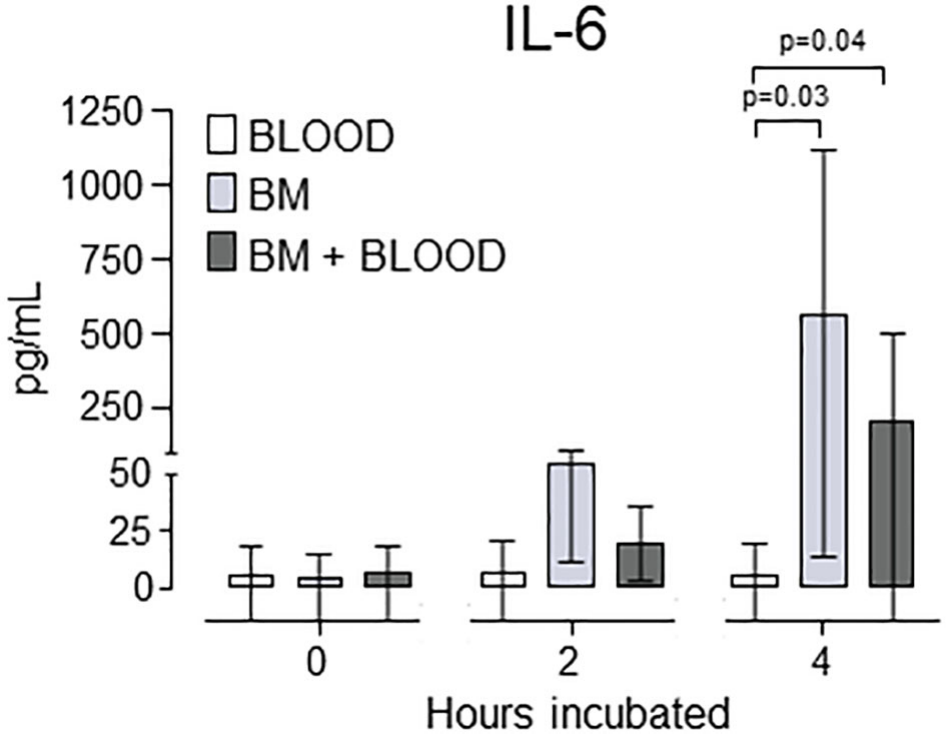
*In vitro* quantification of IL-6. Whole blood (BLOOD), homogenized, homologous bone marrow (BM) and the combination thereof (50-50%) where incubated for up to 4 hours. Data are presented as mean values, with error bars spanning the 95% confidence interval (CI). The p-values are reported from comparing blood alone with bone marrow alone, and blood alone with the combination, respectively, after 4 hours incubation time using the unpaired t-test.

Since IL-6 turned out to be the only marker responding to intramedullary nailing systemically in plasma, porcine whole blood alone, whole blood mixed with 50% autologous bone marrow, and bone marrow alone, were incubated for 0, 120 and 240 minutes and analyzed for IL-6 (**Figure 5**). After 4 hours incubation at 37°C, IL-6 was highest in bone marrow alone at 568 (95% CI 14 - 1122) pg/mL vs 6 (95% CI 0 -19) pg/mL in blood alone (p=0.03) and 212 (95% CI 0 - 505) pg/mL in blood mixed with 50% bone marrow (p=0.04).

## Discussion

In this study, we found that intramedullary nailing of the femur triggered an inflammatory response characterized by a selective increase in IL-6 in plasma, and significantly elevated levels of C3a, TCC, TNF, IL-1β, IL-6 or IL-10 in lung and heart tissue, compared to sham. Thus, intramedullary nailing appeared to elicit an inflammatory response that was hardly detected in plasma samples, where local tissue inflammation was not reflected. Consequently, isolated measurement of these inflammatory markers in plasma may underestimate the whole-body inflammatory response offset by bone marrow emboli. Notably, the intramedullary nailing with release of bone marrow emboli triggered a local inflammatory response that was comparable in intensity to that observed in cases of polytrauma (21), venous air embolism (16) and sepsis (22).

Intravenous injection of bone marrow resulted in increased TAT after 30 minutes and increased plasma IL-6 after 300 minutes, indicating that bone marrow constituents may elicit potent procoagulant and prothrombotic effects (23). The increase in IL-6 was only 5-fold compared to a 21-fold increase in intramedullary reamed pigs, a difference that may be explained by different experimental conditions in the two groups, e.g. that aspirated bone marrow is qualitatively and quantitatively distinct from that released during intramedullary nailing of the femur.

After trauma or surgery, plasma IL-6 increases one hour post-event and reaches its peak approximately six hours later, though it can continue to increase in severe clinical courses (24, 25). Similarly, in our study, we observed increasing IL-6 levels, with the highest levels approximately 5 hours after surgery or bone marrow injection. IL-6 has a longer half-life in plasma than other cytokines like IL-1β and TNF, making it both a marker for and a mediator of inflammation (26). Whether IL-6 acts as a pro- or anti-inflammatory cytokine depends on the overall immune response (15, 27, 28). It may elicit proinflammatory effects by inducing acute-phase proteins in the liver, maturing B-cells, increasing the production of other pro-inflammatory cytokines, and amplifying local innate immune responses (29).

Damage-associated molecular patterns induce the production of IL-6 through several mechanisms, including involvement of the complement system (30). IL-6 plays a role in normal homeostasis and cell differentiation and is produced not only by stimulated macrophages and monocytes but also by bone marrow-derived mesenchymal cells, endothelial cells, and fibroblasts (15). Therefore, both tissue damage in itself and bone marrow release can contribute to elevated IL-6 levels, as certain bone marrow cells produce IL-6 locally in the marrow under normal conditions (1, 2). We found that bone marrow alone released high levels of IL-6 as compared to normal blood when incubated *in vitro*. Bone marrow derived cells are known to contain IL-6, among other cytokines, in humans (31) and pigs (32). When this marrow embolizes to tissues including the heart and lungs, it can be assumed that it contributes to tissue inflammation, which is in accordance with our findings of increased complement activation and cytokines, particularly in the lungs.

Only more extensive orthopedic surgery including femoral nailing significantly increases plasma IL-6, and IL-6 is further elevated if the surgery is performed in multi-trauma patients (33, 34). This can be explained by the fact that both tissue damage itself and bone marrow alone have the potential to increase IL-6 in plasma. Therefore, injuries or procedures causing both greater tissue damage and bone marrow embolism may increase IL-6. As IL-6 seems to become harmful at high plasma concentrations, it may be prudent to perform major surgery only after the initial inflammatory response induced by the primary injury has subsided, whenever clinically possible (35, 36). Delaying definitive surgery has been shown to improve outcome and reduce IL-6 (37–39). Other measures following orthopedic trauma – such as treatment with corticosteroids (40) or technical, intraoperative measures (41), have theoretical but still clinically unproven ameliorating effects.

Intramedullary nailing of the femur often leads to bone marrow emboli, but only occasionally causes fat embolism syndrome (42–44). Identifying risk factors including polytrauma, high lactate levels, and early oxygenation failure therefore becomes crucial (45). Further, the combined effects of multiple traumas and total embolic load likely increases risk of fat embolism syndrome. While the inflammatory impact of femoral nailing, particularly in polytrauma cases, is not extensively described, high IL-6 levels post-injury are associated with elevated risk of fat embolism syndrome (46). Early IL-6 measurement combined with clinical indicators may help identify patients at risk.

As mentioned, bone marrow emboli consist of cell-rich (red) and fat-rich (white) bone marrow, and bone marrow cells such as mesenchymal stem cells and hematopoietic cells can produce, among others, IL-6 and TNF (1–3). White bone marrow also contains cytokines, known as adipokines (47). It has been shown that adipokines like leptin and IL-17A may also increase the risk of multiorgan failure in trauma patients (48). This may contribute to bone marrow emboli eliciting inflammation in the tissue they end up in. Different organs have varying capacities to bind and produce cytokines, and the inflammatory response to bone marrow emboli can differ across organs.

In fat embolism syndrome, respiratory failure is the most common presentation (7), making it relevant to map the inflammatory response in the lungs. The lungs are subjected to inflammation, in part, because the white, fat-rich portion of the bone marrow emboli is broken down in the lungs by lipases into pro-inflammatory, free fatty acids, which cause increased TNF and IL-6 locally in lung tissue due to endothelial damage (41).

Among known mediators of inflammation in the lungs, we found that C3a, TNF and IL-1β were increased after intramedullary nailing. The anaphylatoxins C3a and C5a are associated with lung damage and a local cytokine storm (49, 50) and hypoxia is associated with increased TNF in lung tissue (51). The pigs in our study developed hypoxia shortly after intramedullary nailing, and we detected bone marrow emboli in the lungs in all operated pigs. An explanation for the hypoxia may be that the emboli caused pulmonary capillary obstruction and reduced perfusion, but possibly also reduced diffusion capacity due to inflammation.

Activation of the complement system can stimulate the production of IL-6 when C3a and C5a bind to their receptors (52–54). The role of the complement system in bone marrow embolization or fat embolism syndrome is not well understood. It has not been shown that intramedullary nailing alone causes complement activation, but shown that increased TCC correlates with the Injury Severity Score (ISS) and that TCC is associated with increased mortality in multi-trauma patients (55). One study found complement activation in pigs subjected to polytrauma, which was higher in pigs that additionally underwent intramedullary nailing of the femur (56). As in our study, this study found increased plasma IL-6 and increased C3a in heart tissue. Another porcine study on femoral intramedullary nailing also found increased plasma IL-6 (57).

Among several limitations in this study, the group sizes of both sham (n=4) and of pigs exposed to intravenous bone marrow (n=4) was small compared to the group exposed to intramedullary reaming (n=12). This impacts the power of our results and their significance. However, power calculation found 4 sham animals to be sufficient. Further, exposing four pigs to intravenous bone marrow was conducted as an experimental understudy to understand the impact of bone marrow alone. Although further experiments may be warranted, we did not find it reasonable to sacrifice further animals within this study protocol in concordance with the RRR principles of animal experimentation. Further, the pigs developed transient myocardial ischemia, and it cannot be excluded that this contributed to the findings in plasma or myocardial tissue.

In conclusion, intramedullary femoral nailing induced a selective systemic increase in IL-6 as measured in plasma, in contrast to a broad inflammatory response including complement activation and cytokine release locally. Thus, bone marrow emboli might contribute to a substantial local inflammatory response with organ damage in vital organs like lung and heart, not reflected by blood analyses.

## Data availability statement

The raw data supporting the conclusions of this article will be made available by the authors, without undue reservation.

## Ethics statement

The study was approved by the Norwegian Animal Welfare Committee (FOTS ID 19803) and conducted in accordance with the Norwegian regulations for laboratory animal care and EU directive 2010/63/EU. The studies were conducted in accordance with the local legislation and institutional requirements. Written informed consent was obtained from the owners for the participation of their animals in this study.

## Author contributions

SK: Methodology, Investigation, Data curation, Conceptualization, Validation, Writing – review & editing, Supervision, Resources. BS: Validation, Resources, Project administration, Methodology, Funding acquisition, Data curation, Writing – review & editing, Supervision, Investigation, Formal analysis, Conceptualization. ÅE: Writing – review & editing, Resources, Methodology, Data curation. RG: Writing – review & editing, Methodology, Formal analysis, Data curation. KP: Writing – review & editing, Methodology, Formal analysis, Data curation. JH: Writing – review & editing, Investigation, Data curation. AJ: Writing – review & editing, Methodology, Investigation, Conceptualization. ML-O: Writing – review & editing, Methodology, Investigation, Data curation. SN: Writing – review & editing, Investigation, Data curation. BN: Writing – review & editing, Methodology, Investigation, Data curation. EN: Software, Project administration, Funding acquisition, Conceptualization, Supervision, Resources, Methodology, Data curation, Writing – review & editing, Investigation. TM: Validation, Supervision, Resources, Project administration, Methodology, Investigation, Funding acquisition, Data curation, Conceptualization, Writing – review & editing.
